# Pregnancy Outcomes and Blood Pressure Visit-to-Visit Variability and Level in Three Less-Developed Countries

**DOI:** 10.1161/HYPERTENSIONAHA.120.16851

**Published:** 2021-03-29

**Authors:** Laura A. Magee, Jeffrey Bone, Salwa Banoo Owasil, Joel Singer, Terry Lee, Mrutunjaya B. Bellad, Shivaprasad S Goudar, Alexander G. Logan, Salésio E. Macuacua, Ashalata A. Mallapur, Hannah L. Nathan, Rahat N. Qureshi, Esperança Sevene, Andrew H. Shennan, Anifa Valá, Marianne Vidler, Zulfiqar A. Bhutta, Peter von Dadelszen

**Affiliations:** 1 Department of Women and Children’s Health, School of Life Course Sciences, Faculty of Life Sciences and Medicine (L.A.M., H.L.N., A.H.S., P.v.D.), King’s College London, United Kingdom.; 2 GKT School of Biomedical Sciences (S.B.O.), King’s College London, United Kingdom.; 3 Department of Obstetrics and Gynaecology, BC Children’s Hospital Research Institute (L.A.M., J.B., M.V., P.v.D.); 4 Centre for Health Evaluation and Outcome Sciences, Providence Health Care Research Institute (J.S., T.L.), University of British Columbia, Vancouver, Canada.; 5 KLE Academy of Higher Education and Research’s J N Medical College, Belagavi, Karnataka, India (M.B.B., S.S.G.).; 6 Department of Medicine, University of Toronto, Canada (A.G.L.).; 7 Centro de Investigação em Saúde de Manhiça, Manhiça, Mozambique (S.E.M., E.S., A.V.).; 8 S Nijalingappa Medical College, Hanagal Shree Kumareshwar Hospital and Research Centre, Bagalkote, Karnataka, India (A.A.M.).; 9 Centre of Excellence, Division of Woman and Child Health, Aga Khan University, Karachi, Pakistan (R.N.Q., Z.A.B.).; 10 Department of Physiological Sciences, Clinical Pharmacology, Faculdade de Medicina, Universidade Eduardo Mondlane, Maputo, Mozambique (E.S.).; 11 Centre for Global Child Health, Hospital for Sick Children, Toronto, Canada (Z.A.B.).

**Keywords:** blood pressure, morbidity, mortality, preeclampsia, pregnancy

## Abstract

In pregnancy in well-resourced settings, limited data suggest that higher blood pressure (BP) visit-to-visit variability may be associated with adverse pregnancy outcomes. Included were pregnant women in 22 intervention clusters of the CLIP (Community-Level Interventions for Preeclampsia) cluster randomized trials, who had received at least 2 prenatal contacts from a community health worker, including standardized BP measurement. Mixed-effects adjusted logistic regression assessed relationships between pregnancy outcomes and both BP level (median [interquartile range]) and visit-to-visit variability (SD and average real variability [ARV], adjusted for BP level), among all women and those who became hypertensive. The primary outcome was the CLIP composite of maternal and perinatal mortality and morbidity. Among 17 770 pregnancies, higher systolic and diastolic BP levels were associated with increased odds of the composite outcome per 5 mm Hg increase in BP (odds ratio [OR], 1.05 [95% CI, 1.03–1.07] and OR, 1.08 [1.06–1.11], respectively). Higher BP visit-to-visit variability was associated with increased odds, per a SD increase in BP variability measure, of (1) hypertension (systolic: OR, 2.09 [1.98–2.21] for SD and 1.52 [1.45–1.60] for ARV; diastolic: OR, 2.70 [2.54–2.87] for SD and 1.86 [1.76–1.96] for ARV); and (2) the composite outcome (systolic: OR, 1.10 [1.06–1.14] for SD and 1.06 [1.02–1.10] for ARV; diastolic: OR, 1.07 [1.03–1.11] for SD and 1.06 [1.02–1.09] for ARV). In 3 less-developed countries, higher BP level and visit-to-visit variability predicted adverse pregnancy outcomes, providing an opportunity for high-definition medicine.

## Abstract 2

Supplemental Digital Content is available in the text.

Hypertension in pregnancy is a systolic blood pressure (BP) of 140 mm Hg or higher and diastolic BP of 90 mm Hg or higher.^1^ There is a continuous relationship between higher BP and worse maternal outcomes,^2–5^ but severe pregnancy hypertension (systolic BP of at least 160 mm Hg or diastolic BP of at least 110 mm Hg), in particular, is associated with elevated maternal and perinatal risk.^6^

Outside pregnancy, BP level^7^ and its oscillations between measurements (ie, variability) are associated with cardiovascular risk.^8^ In a high-quality review of 19 observational cohort studies and 17 clinical trial cohorts, higher long-term systolic BP visit-to-visit variability in office settings was associated with higher: all-cause mortality (hazard ratio, 1.15 [95% CI, 1.09–1.22]), cardiovascular disease mortality (1.18 [1.09–1.28]), cardiovascular disease events (1.18 [1.07–1.30]), coronary heart disease (1.10 [1.04–1.16]), and stroke (1.15 [1.04–1.27]).^8^ BP variability reflects the integrated impact of environment (eg, noise), behavior (eg, lifestyle), and biology (eg, intrinsic arterial and cardiopulmonary reflexes).^9^

As in nonpregnant individuals, BP in pregnancy varies over a 24-hour period, with the pattern associated with hypertensive disease onset.^10^ BP variability may also moderate adverse pregnancy outcomes. In a secondary analysis of CHIPS trial data (Control of Hypertension In Pregnancy Study, URL: https://www.clinicaltrials.gov; Unique identifier: NCT01192412)^11^ from outpatient women with chronic or gestational hypertension, higher BP level predicted adverse events.^12^ While higher BP visit-to-visit variability (adjusted for BP level) was associated with increased odds of severe hypertension and preeclampsia; greater diastolic BP visit-to-visit variability was associated with fewer adverse perinatal outcomes, suggesting a possible fetal advantage of variability.

To enable high-definition medicine in less-developed settings, we studied the relationship between pregnancy outcomes and both BP level and long-term visit-to-visit variability in the CLIP (Community-Level Interventions in Preeclampsia) cluster randomized trials (URL: https://www.clinicaltrials.gov; Unique identifier: NCT01192412).^13–16^

## Methods

This was an unplanned secondary analysis of data from the 22 intervention clusters of the CLIP cluster randomized trials, aimed at externally validating findings from the CHIPS trial.^12^ Data can be accessed through the CLIP trials data access committee (Text S1 in the Data Supplement).

### The CLIP Trials

The CLIP trials were conducted in 2014 to 2017 in India (N=6 intervention clusters), Pakistan (N=10), and Mozambique (N=6).^13–16^ The unit of randomization (cluster) was the local administrative unit.

All pregnant women aged 15 to 49 years (12–49 years in Mozambique) were identified in their community by trained community health workers. All women provided written informed consent to participate. The trial was unmasked given the nature of the intervention, aimed at addressing the 3 delays in triage, transport, and treatment related to preeclampsia. First, community engagement addressed barriers and facilitators to accessing care. Second, existing cadres of community health workers were trained to task-share pregnancy hypertension-oriented care at CLIP contacts in women’s homes, using the CLIP PIERS (Preeclampsia Integrated Estimate of Risk Score) on-the-Move (POM) digital health application for risk stratification.^17^

Community health workers (1) responded to emergency conditions, if relevant; (2) took women’s BP and assessed dipstick proteinuria at the first and any hypertensive contact; (3) administered oral methyldopa 750 mg for BP of at least 160/110 mm Hg; (4) administered 10 g intramuscular magnesium sulfate for suspected severe preeclampsia; and (5) and referred to a comprehensive emergency obstetric care facility.

Standardized BP measurement by trained community health workers used a semiautomated pregnancy- and preeclampsia-validated oscillometric device (Microlife 3AS1-2).^18^ Having rested for 5 minutes, women’s BP was measured at least twice; all readings were entered into the POM application, which averaged the first and second readings and if they differed by >10 mm Hg, a third reading was requested and the second and third readings averaged. The planned frequency of prenatal POM-guided CLIP contacts was every 4 weeks, at minimum.

Trained surveillance teams conducted regular surveys of households (every 3–6 months), except in India where a prospective population-based surveillance system was established. The primary outcome was a composite of all-cause maternal and perinatal mortality and morbidity. Maternal death or morbidity occurred during or within 42 days of pregnancy; morbidity was one or more life-threatening complications of pregnancy, defined as a serious end-organ complication of preeclampsia (eg, eclampsia), another major cause of maternal mortality/morbidity (ie, obstetric sepsis or vaginal fistula), or receipt of a life-saving intervention. Perinatal death was stillbirth, early or late neonatal mortality, and morbidity a composite of problems that could be ascertained in community (eg, seizure or feeding difficulty). For detailed definitions, see Table S2 in the Data Supplement.

Overall coordination and data management were by the Preeclampsia–Eclampsia Monitoring, Prevention and Treatment research group at the University of British Columbia, Canada. Ethical approvals were granted by the University of British Columbia (H12-03497) and relevant in-country research ethics boards (Aga Khan University, Pakistan, 2590-Obs-ERC-13; KLE University, India, MDC/IECHSR/2011-12/A-4, ICMR 5/7/859/12-RHN; and Centro de Investigação em Saúde de Manhiça (CIBS-CISM/038/14) and Mozambique National Bioethic Committee (219/CNBS/14). The trials are registered at URL: https://www.clinicaltrials.gov (Unique identifier: NCT01911494) and the related individual participant data meta-analysis on PROSPERO (CRD42018102564).

### BP Level and Variability

We included CLIP participants in pregnancy, from enrollment until follow-up for the CLIP primary outcome, who had at least 2 antenatal contacts by community health workers (prerequisite for determining BP variability, see below).

Mean systolic and diastolic BP levels were the mean of relevant values taken at all POM-guided CLIP contacts between enrollment and delivery.

Within-participant visit-to-visit BP variability was assessed using all POM-guided CLIP contacts after enrollment until delivery. We evaluated 2 measures of BP visit-to-visit variability used outside pregnancy: (1) within-participant SD to reflect dispersion of measurements around mean BP and (2) average real variability (ARV) as the average of the absolute successive difference of all BP values, reflecting changes over short time intervals (so a decrease by 4 mm Hg and then an increase by 6 mm Hg would represent an ARV of 5). We adjusted for mean BP level, as higher levels are associated with more variability. Any correlation was explored between BP variability and number of measurements.

### Statistical Analyses

In our primary analysis, relationships were explored between each major CLIP outcome and both BP level and visit-to-visit variability, using values before the outcomes: progression to hypertension (systolic BP of at least 140 mm Hg or a diastolic BP of at least 90 mm Hg, based on an average of 2 measurements), composite of maternal or perinatal mortality or morbidity (primary outcome), composite maternal outcome (mortality or morbidity) and composite perinatal outcome (stillbirth, early or late neonatal death, or neonatal morbidity) to evaluate whether the direction of effect on maternal outcomes was the same. In addition, we further examined the relationship between each major CLIP outcome and BP variability only among women who became hypertensive to see if BP variability could add further information to BP level.

Data were summarized as median and interquartile range and counts (percentages) for continuous and categorical variables, respectively.

The mean BP level-outcome relationship was explored by mixed-effects logistic regression. Adjustment was made for country and cluster (each as a random effect), maternal age at enrollment, maternal education, parity, and gestational age at enrollment. The odds ratio (OR) for each outcome was calculated per 5 mm Hg increase in mean BP from enrollment until delivery.

The BP variability-outcome relationship was evaluated for all women, and specifically for women who developed pregnancy hypertension, by mixed-effects logistic regression, adjusted for average BP level (defined as the mean of the BP readings used to define visit-to-visit variability) and the variables described above for BP level analyses. The change in the scale of the OR was calculated per SD increase in both metrics of BP variability to compare the relative importance of one measure with another. Correlation between BP visit-to-visit variability and the number of measurements was assessed by Spearman correlation (*r*).

In sensitivity analyses: (1) for all outcomes, we excluded BP values within 7, 14, 21, and 28 days before delivery to minimize the extent to which BP variability may be an artifact of the outcomes themselves (ie, reverse causality); (2) for all outcomes, we added further adjustment for the final antenatal BP measurement, to account for BP trajectory; (3) for all outcomes, we excluded repeat pregnancies for the same woman; and (4) for progression to hypertension, we incorporated diagnoses based only on trial surveillance data for women who became hypertensive after their last POM-guided visit. A *P*<0.05 was considered statistically significant, without adjustment for multiple comparisons.

All statistical analyses were performed using and R 3.5.3 (R Development Core Team, Vienna, Austria). J. Bone had access to all data and takes responsibility for its integrity and the data analysis.

## Results

### Patients

**Figure 1.**
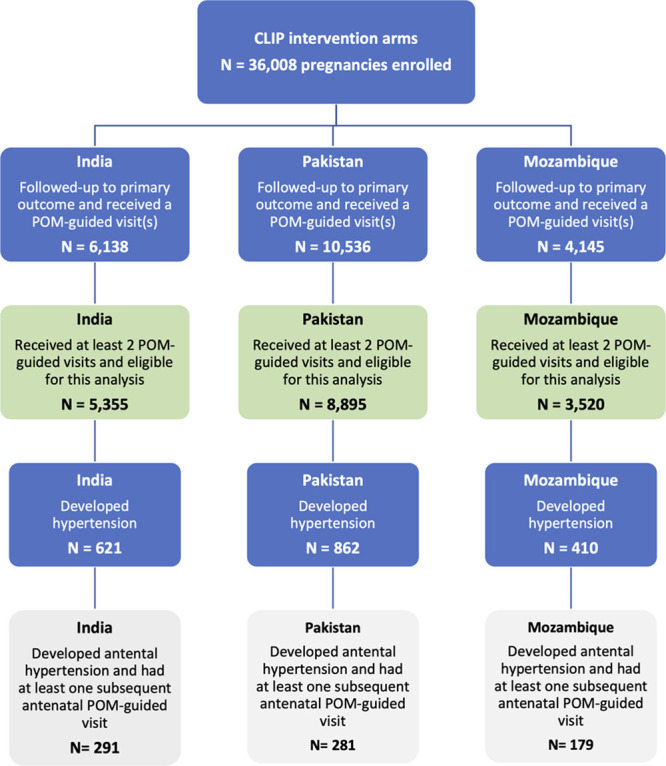
**Inclusion of participants from each of 3 CLIP trials (Community-Level Interventions in Preeclampsia).** POM indicates PIERS (Preeclampsia Integrated Estimate of Risk Score) on-the-Move

Of 36 008 pregnancies enrolled in CLIP intervention clusters, 20 819 were followed-up to the primary outcome and had at least one POM contact,^13–16^ of whom 17 770 pregnancies had at least 2 antenatal POM-guided contacts and were eligible for this analysis (green boxes, Figure 1). Approximately half (9534, 53.6%) of women had POM-guided CLIP visits at least monthly. Hypertension developed in 1893 (10.7%) of pregnancies, and 751 developed hypertension antenatally and had at least one subsequent antenatal POM-guided visit (gray boxes, Figure 1).

Some baseline maternal characteristics and outcomes differed between countries (Table). On average, women were older (late 20s) in Pakistan, enrolled earliest in pregnancy in India and latest in Mozambique, and most were parous. Slightly more than half of Indian and Mozambican, but about a third of Pakistani, women had a basic education. On average, women had vaginal births at term, in facility. The primary composite outcome occurred in approximately one-quarter of pregnancies, with at least half related to perinatal mortality or morbidity. Women eligible for these analyses were representative of the study population with regards to baseline characteristics and outcomes (Table S3).

**Table.**
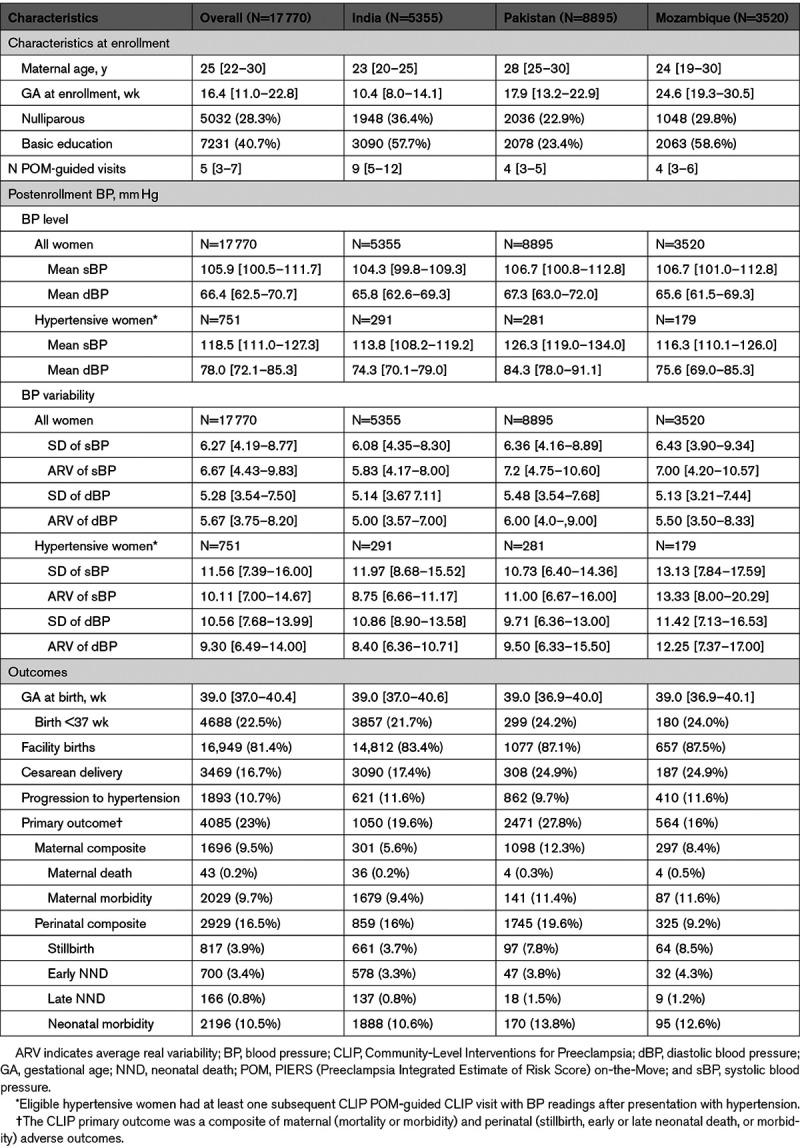
Characteristics of All Included CLIP Pregnancies by Country (Median [Interquartile Range] and N (%), Unless Otherwise Specified)

### BP Level

**Figure 2.**
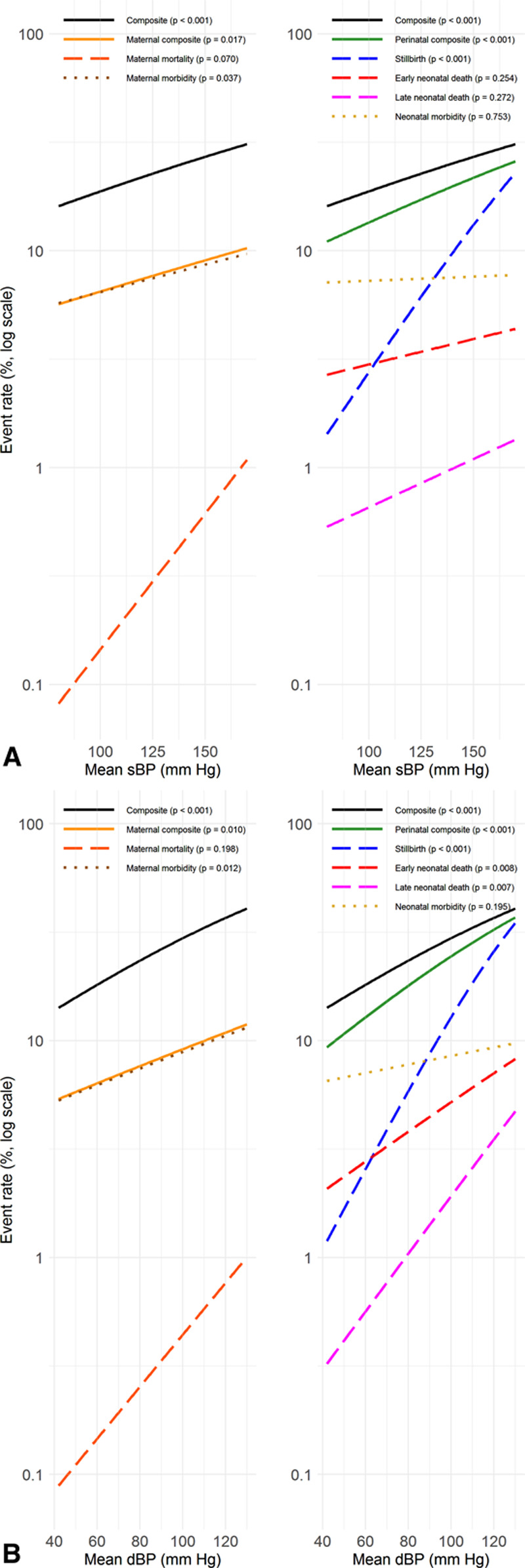
**Relationship among all pregnant women, between adverse pregnancy outcomes and higher systolic and diastolic blood pressure (sBP and dBP) levels.** **A**, Adverse outcomes and sBP. **B**, Adverse outcomes and dBP. The results are adjusted for country and cluster (as random effects), maternal age at enrollment, maternal education, parity, gestational age at enrollment, and mean BP level.

Among all women, mean systolic and diastolic BP levels were similar between countries (Table). Higher systolic (Figure 2A) and diastolic (Figure 2B) BP levels were associated with increased odds of developing the composite CLIP primary outcome and its components for all but neonatal morbidity (Table S4).

### BP Variability and Outcomes

BP variability, assessed by SD or ARV, was similar between countries, for all women and for those who developed hypertension. There was no relationship between the number of BP measurements and variability, measured by SD (systolic BP *r*=0.078, diastolic BP *r*=0.079) or ARV (systolic BP *r*=−0.117, diastolic BP *r*=−0.18). Likewise, there was no relationship between the number of BP measurements per week enrolled in trial and any of the variability measures (*r*<0.001 in all cases).

**Figure 3.**
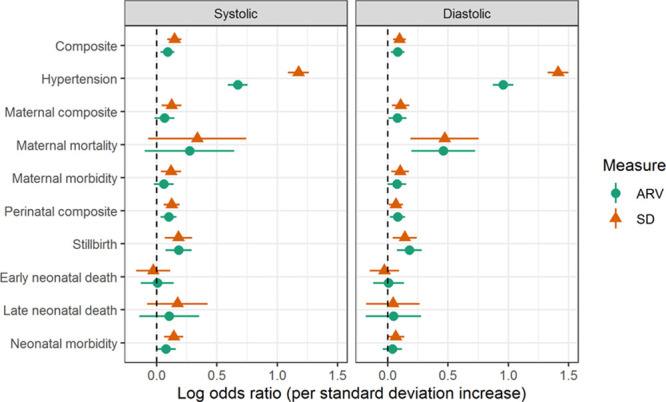
Relationship among all pregnant women, between adverse pregnancy outcomes and higher systolic and diastolic blood pressure (BP) variability*. The results are adjusted for country and cluster (as random effects), maternal age at enrollment, maternal education, parity, gestational age at enrollment, and mean BP level. ARV indicates average real variability.

Among all women, higher BP visit-to-visit variability (adjusted for country and cluster as random effects, and maternal age at enrollment, maternal education, parity, gestational age at enrollment, and mean BP level) was associated with increased odds of developing hypertension, the CLIP primary outcome, the maternal and perinatal composites, and most of their components (Figure 3; Table S5), including maternal mortality and stillbirth. The findings were evident for systolic and diastolic BP and both measures of variability.

Sensitivity analyses that removed BP values 7 or more days before delivery showed the association between BP variability and outcomes was largely lost for systolic BP but retained for diastolic BP for maternal death and stillbirth; there was little change in relationships between variability and perinatal outcomes (Table S6). Further adjustment for the last BP before delivery slightly attenuated the association between BP variability and maternal outcomes, but there was no change for perinatal outcomes (Table S7). Exclusion of the 926 repeat pregnancies left the outcomes unchanged (Table S8). When progression to hypertension included diagnoses from trial surveillance, its relationship with BP variability remained highly significant (*P*<0.001; Table S9).

**Figure 4.**
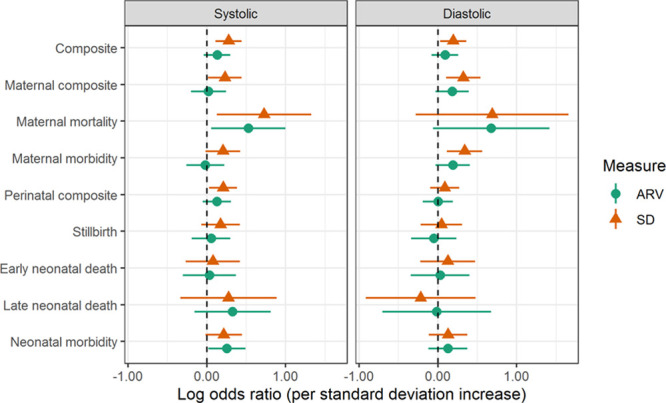
**Relationship among hypertensive pregnant women, between adverse pregnancy outcomes and higher systolic and diastolic blood pressure (BP) variability.** The results are adjusted for country and cluster (as random effects), maternal age at enrollment, maternal education, parity, gestational age at enrollment, and mean BP level. ARV indicates average real variability.

Among women who became hypertensive before birth, from that point onward in pregnancy, higher BP variability was associated with increased odds of the CLIP primary outcome, as well as the maternal composite, maternal mortality, maternal morbidity, and perinatal morbidity (Figure 4; Table S10); this was especially true of systolic BP variability. Planned sensitivity analyses were not possible as too few women who became hypertensive remained in their communities and had ongoing POM-guided CLIP visits.

## Discussion

In CLIP intervention clusters in resource-limited settings, higher postenrollment BP level and visit-to-visit variability (adjusted for BP level) from standardized measurements was associated with more adverse maternal and perinatal outcomes. Prediction of hypertension was particularly good for BP variability based on SD rather than ARV. However, there was evidence that particularly for maternal outcomes, at least some of the BP variability detected was an early part of the outcome (and may enable early response). A similar (particularly for systolic BP visit-to-visit variability), albeit attenuated, pattern of effect with outcomes was seen among women who developed hypertension.

In this era of electronic health records, we envision that BP variability could be computed and incorporated into personalized maternity care and as a woman’s BP values are evaluated on an ongoing basis at each antenatal care contact. Of note, as outlined in the results, there was no relationship between the number of BP measurements and variability (measured by SD or ARV) or the number of BP measurements per week women were enrolled in the trial and any of the variability measures. While our observations are important clinically, introduction into clinical care would require establishment of normative ranges associated with the absence of adverse maternal and perinatal outcomes associated with pregnancy hypertension or other complications that derive from placental dysfunction (eg, abruption or stillbirth). Our findings should encourage this, as the CLIP trials illustrated that the computing power of digital technology, in the hands of those outside the traditional medical model (and therefore, potentially, women themselves), can be harnessed to achieve personalized medicine.^19^ The limitation is human resource capacity, which in the CLIP trials, related to use of the existing workforce, for scalability.

There is a known continuous relationship between higher BP and more adverse maternal outcomes, regardless of the hypertensive disorder of pregnancy.^2,3,5^ Our data confirm this relationship in resource-constrained settings. This is true even when accounting for treatment goals and antihypertensive therapy^20^ and has led to calls for use in pregnancy of the American College of Cardiology/American Heart Association cutoff of 130/80 mm Hg for stage 1 hypertension.^21^

To our knowledge, ours is the first article to report the relationship between visit-to-visit BP variability and pregnancy outcomes among both unselected and hypertensive pregnancy, and in resource-limited settings. Specifically, we have shown a relationship between visit-to-visit BP variability and maternal mortality and stillbirth. Our findings extend the results of limited publications.

Among unselected pregnancies in China (N=14 702), long-term visit-to-visit BP variability (by SD and coefficient of variation and adjusted for BP level and important covariates) was predictive of gestational hypertension and preeclampsia^22^; systolic variability was most strongly related to development of hypertension, in contrast to systolic and diastolic variability that were predictive among CLIP participants in South Asia and sub-Saharan Africa. Other pregnancy outcomes were not reported, and the impact not presented of excluding almost half of women with fewer than 3 visits in each of the second and third trimesters. In a nested case-control study (484 hypertensive and 3679 normotensive controls matched by propensity score), higher BP visit-to-visit variability, adjusted for BP level, was associated with more maternal and fetal complications.^23^ Neither study formally examined the impact of including BP measurements close to delivery (or occurrence of the outcome).

Among women with hypertension in the CHIPS trial^12^; higher visit-to-visit BP variability (adjusted for BP level and covariates) was associated with more adverse maternal outcomes (ie, severe hypertension and progression to preeclampsia), with evidence that the relationship was based, at least in part, on BP values close to delivery, as seen in our CLIP data for the maternal mortality and morbidity composite. However, higher BP variability may have been associated with improved perinatal outcomes, particularly for diastolic BP, in contrast to CLIP. First, CHIPS was undertaken primarily in more-developed country sites where fetal surveillance and neonatal care were routinely available, compared with rural Asian and African communities in CLIP. The alternative explanation that higher BP variability in CHIPS may have improved uteroplacental perfusion is not supported by our CLIP findings. Second, CHIPS treated to a target diastolic BP which may have led to differences not seen in CLIP with regards to associations between systolic and diastolic variability and outcomes.

The mechanism is unknown for the relationship between visit-to-visit BP variability and either cardiovascular risk outside pregnancy or adverse pregnancy outcomes. The pathophysiology proposed has included arterial remodeling and antihypertensive agent (with calcium channel blockers associated with less variability),^24^ as well as environmental and behavioral influences (such as lack of sleep), and cardiovascular homeostasis.^25^ Whether the mechanisms are the same outside and in pregnancy is also unknown.

Our major strengths are population-based recruitment, large sample size, standardized BP measurement using a pregnancy-validated device,^18^ analysis of unselected and hypertensive pregnancies without selection for timing of BP measurements and accounting for the impact of measurements close to birth and reporting of preeclampsia and mortality and morbidity. We used accepted measures of BP variability, could not identify a relationship between the frequency of BP measurement and BP variability, and adjusted for BP level and relevant baseline characteristics.

Limitations include community-only BP values. As published, community health workers were unable to provide the protocol-specified frequency of contacts, particularly the weekly visits from 36 weeks, so we may have missed some term-onset hypertension.^26^ Once hypertensive, the minority of women continued with care in their communities; this, plus presumed use of antihypertensive therapy, may have either limited our power to examine the BP variability-outcome relationship among women with hypertension or attenuated that relationship. Basic maternal characteristics were available for our adjusted analyses and as many women booked only after 20 weeks, some with chronic hypertension (1%–2% of pregnancies) may have been unrecognized as previously hypertensive. We did not have details about antihypertensives used once hypertension was diagnosed; while nifedipine has been associated with less 24-hour BP variation than labetalol in women with chronic hypertension,^27^ nifedipine is uncommonly used for hypertension in Countdown 2030 countries. No biological samples were collected to enable exploration of other pathophysiological pathways underlying the BP variability-outcome relationships. Finally, while emphasizing 95% CI to identify associations of potential interest, multiple comparisons were made.

## Perspectives

In pregnancy, both higher BP level and variability are adverse prognostic markers for mothers and babies in resource-constrained settings. The increasing use of digital technology in global health care now provides us with the opportunity to harness the information provided by other aspects of BP beyond level, to provide high-definition medicine to those pregnant women most at risk.

## Acknowledgments

The secondary analyses were conceived by L.A. Magee and P. von Dadelszen, with an active contribution by J. Bone, T. Lee, and J. Singer. J. Bone performed the analyses. All authors participated in the interpretation of the results, their interpretation and write-up, and approved the final version of the article.

## Sources of Funding

This trial was funded by the University of British Columbia, a grantee of the Bill & Melinda Gates Foundation (OPP1017337).

## Disclosures

None.

## Supplementary Material



## Appendix

### CLIP Trials Study Group

CLIP Trials Working Group: Esperança Sevene, Eusébio Macete, Khátia Munguambe, Charfudin Sacoor, Anifa Vala, Helena Boene, Felizarda Amose, Rosa Pires, Zefanias Nhamirre, Marta Macamo, Rogério Chiaú, Analisa Matavele, Faustino Vilanculo, Ariel Nhancolo, Silvestre Cutana, Ernesto Mandlate, Salésio Macuacua, Cassimo Bique, Sibone Mocumbi, Emília Gonçálves, Sónia Maculuve, Ana Ilda Biz, Dulce Mulungo, Orvalho Augusto, Paulo Filimone, Vivalde Nobela, Corsino Tchavana, Cláudio Nkumbula

Rahat Qureshi, Zulfiqar A Bhutta, Zahra Hoodbhoy, Farrukh Raza, Sana Sheikh, Javed Memon, Imran Ahmed, Amjad Hussain

Mrutunjaya B Bellad, Umesh S Charantimath, Shivaprasad S Goudar, Geetanjali M Katageri, Avinash J Kavi, Amit P Revankar, Ashalata A Mallapur, Umesh Y Ramdurg, Shashidhar G Bannale, Vaibhav B Dhamanekar, Geetanjali I Mungarwadi, Narayan V Honnungar, Bhalachandra S Kodkany, Anjali M Joshi, Uday S Kudachi, Sphoorthi S Mastiholi, Chandrappa C Karadiguddi, Gudadayya S Kengapur, Namdev A Kamble, Keval S Chougala

Jeffrey Bone, Dustin T Dunsmuir, Sharla K Drebit, Chirag Kariya, Mai-Lei Woo Kinshella, Tang Lee, Jing Li, Mansun Lui, Beth A Payne, Diane Sawchuck, Sumedha Sharma, Domena K Tu, Marianne Vidler, Ugochi V Ukah, Laura A Magee, Peter von Dadelszen

CLIP Trial Steering Committee: J Mark Ansermino, Ana Pilar Betrán, Richard Derman, Shafik Dharamsi, France Donnay, Sharla Drebit, Guy Dumont, Susheela M Engelbrecht, Veronique Fillipi, Tabassum Firoz, William Grobman, Marian Knight, Ana Langer, Simon Lewin, Gwyneth Lewis, Craig Mitton, Nadine Schuurman, Andrew Shennan, Joel Singer, Jim Thornton, Hubert Wong

CLIP Trial Executive Committee: Olalekan O Adetoro, Mrutunjaya M Bellad, Zulfiqar A Bhutta, Peter von Dadelszen, Shivaprasad S Goudar, Laura A Magee, Ashalata A Mallapur, Khátia Munguambe, Beth A Payne, Rahat Qureshi, Charfudin Sacoor, Esperança Sevene, Sumedha Sharma, John O Sotunsa, Marianne Vidler

CLIP Data Safety and Monitoring Board (DSMB): Romano Nkumbwa Byaruhanga, Brian Darlow, Eileen Hutton, Mario Merialdi, Lehana Thabane

## Novelty and Significance

### What Is New?

- This is the first study to consider the relationship between changes in blood pressure levels at different prenatal visits and how they affect pregnancy outcomes. This study is also the first to include both unselected and hypertensive women in low resource settings.

### What Is Relevant?

- In a large study of pregnant women that took place across three countries, both higher blood pressure levels and greater variation in prenatal visit blood pressure measurements were associated with higher rates of adverse maternal and perinatal outcomes. This was the case for both women with normal and high blood pressure levels.

### Summary

Variation in both systolic and diastolic blood pressure measurements between prenatal visits is associated with a progression to high blood pressure. Changes occurring in blood pressure levels during pregnancy are associated with adverse outcomes for both mothers and babies and may be useful as an indicator to provide preventative care to women in need.
