# Supplementary material for: Pregnancy Outcomes and Blood Pressure Visit-to-Visit Variability and Level in Three Less-Developed Countries
Source: Hypertension. 2021 Mar 29;77(5):1714–22. doi: 10.1161/HYPERTENSIONAHA.120.16851 (PMC8284372; doi:10.1161/HYPERTENSIONAHA.120.16851)
Supplement: Supplementary file 1 [file hyp-77-1714-s001.pdf]

DATA SUPPLEMENT

Pregnancy outcomes and blood pressure visit-to-visit variability and level in three less-developed countries

Laura A Magee, MD*

Jeffrey Bone, MSc*

Salwa Banoo Owasil, iBSc

Joel Singer, PhD

Terry Lee, PhD

Mrutunjaya B Bellad, MD

Shivaprasad S Goudar, MD

Alexander G Logan, MD

Salésio E Macuacua, MD

Ashalata A Mallapur, MD

Hannah L Nathan, PhD

Rahat N Qureshi, MBBS

Esperança Sevene, PhD

Andrew H Shennan, MD

Anifa Valá, MVD

Marianne Vidler, PhD

Zulfiqar A Bhutta, PhD

Peter von Dadelszen, DPhil

and The CLIP Study Group

* Joint first authors

Address for correspondence:

Professor Laura A. Magee

Department of Women and Children's Health

School of Life Course Sciences, King's College London

Email: Laura.A.Magee@kcl.ac.uk

SUPPLEMENTARY MATERIAL to Pregnancy outcomes and blood pressure visit-to-visit variability and level in three less-developed countries

	Name	Page
**Text**		
Text S1	Data sharing statement	4
**Tables**		
Table S2	Definition of the CLIP primary outcome components	6
Table S3	Summary of pregnancies included in each analysis	8
Table S4	Relationship amongst all women, between mean systolic and diastolic blood pressure level and CLIP outcomes	9
Table S5	Relationship amongst all women, between systolic and diastolic blood pressure variability and CLIP outcomes	10
Table S6	Sensitivity analyses - all women, relationship between blood pressure variability and outcomes, excluding blood pressure measurements taken 7, 14, 21 and 28 days prior to delivery	11
Table S7	Sensitivity analyses - all women, relationship between blood pressure variability and outcomes, adjusting for the last BP value before birth	13
Table S8	Sensitivity analyses - Relationship amongst all women, between systolic and diastolic blood pressure variability and CLIP outcomes, excluding repeat pregnancies	15
Table S9	Sensitivity analyses – all women, relationship between blood pressure variability and progression to hypertension, incorporating the diagnosis from household surveys and facility records	16
Table S10	Relationship amongst hypertensive women between blood pressure variability and maternal and perinatal outcomes	17

Text S1: CLIP Trials Data Sharing Statement

The CLIP Trial data are de-identified participant-level data. Once the primary CLIP manuscripts, individual participant data meta-analysis, and papers based on the other pre-defined analyses are published as per the Statistical Analysis Plan (SAP), the data will be freely available to academically-active entities (e.g., universities, NGOs, multilaterals), with the CLIP Principal Investigator (Peter von Dadelszen) or named delegate as a named co-investigator, for the purposes of pregnancy-related research and within the limits of the informed consent obtained. Access will be through the CLIP Trials Data Access Committee*, contacted at 'PRE-EMPT@cw.bc.ca', as referenced on our website at 'https://PRE-EMPT.bcchr.ca'. A full data dictionary and all study documents will be available. Access will be through written application. When approved, a quote for the costs of preparing the data will be provided to the applicant.

By submitting an application form, the investigator agrees that s/he has read, understood and agrees to the terms and conditions below:

1. S/he is an academically-active researcher affiliated with an entity able to engage in a data transfer agreement;
2. S/he warrants that the information entered is to the best of her/his knowledge full and correct;
3. S/he agrees that the Data Sharing Agreement will only be used for the specific project outlined in the application;
4. S/he represents that s/he has obtained the necessary approvals to transfer the data and/or receive the data under this Data Sharing Agreement;
5. S/he understands that the responses provided will form part of a legally-binding document;
6. S/he understands that the Agreement is not valid until a fully-executed copy, with signatures from all parties, is emailed to PRE-EMPT@cw.bc.ca); and
7. S/he understands that no modifications can be made to the Data Sharing Agreement and if modifications are made, the Data Sharing Agreement will be rendered invalid.

There is no pregnancy-specific repository for us to access, but once the primary papers for the CLIP Trials have been published, we will be depositing a copy of our data in the HBGDki repository at the Bill & Melinda Gates Foundation, our funder. The p<u>ermitted uses and disclosures of these data are as follows:</u>

1. The Foundation will limit the use and disclosure of the CLIP data to conduct research related to achieving the goals of the Foundation as represented above. The Foundation may also de-identify the data set and aggregate it with other de-identified information.
2. The Foundation will restrict access to the CLIP data to individuals involved in the Foundation's research who have a need to access the CLIP data to carry out their duties as they relate to the Permitted Uses and Disclosures identified above, and any such access will be consistent with the assurances and obligations set forth in this Agreement. The Foundation will use appropriate safeguards to prevent use or disclosure of the CLIP data other than as permitted by this Agreement.
3. The Foundation will report to HBGDki Collaborator any use or disclosure of the CLIP data not provided for by this Agreement of which the Foundation becomes aware.
4. The Foundation will ensure that any agents, including subcontractors, to whom it provides the CLIP data, if any, agree to the same restrictions and conditions that apply to the Foundation with respect to such information.

** The **Data Access Committee** is made up of the following individuals: Peter von Dadelszen and Laura A. Magee (King's College London, UK); Zulfiqar A Bhutta (Aga Khan University, Karachi, Pakistan and the Hospital for Sick Children, Toronto, Canada); Rahat N Qureshi (Aga Khan University, Karachi, Pakistan); Ashalata A Mallapur (S Nijalingappa Medical College, Bagalkote, India); Mrutyunjaya B Bellad and Shivaprasad Goudar (KLE Academy of Higher Education and Research's JN Medical College, Belagavi, India); Khátia Munguambe, Charfudin Sacoor, and Esperança Sevene (Centro de Investigação em Saúde da Manhiça, Manhiça, Mozambique)*

Table S2: CLIP primary outcome components

CLIP primary outcome components	Definition
**MATERNAL**	
**Maternal death**	Number of deaths during pregnancy or within 42 days of pregnancy (or last contact day if contact not maintained to 42 days) per 1,000 identified pregnancies, termed Maternal Death Rate
**Maternal morbidity**	Number of women with one or more life-threatening complications of pregnancy during pregnancy or within 42 days of pregnancy or last contact day if contact not maintained to 42 day per 1,000 identified pregnancies. These are the serious end-organ complications of pre-eclampsia, other major causes of maternal mortality, or life-saving interventions related to one of the aforementioned.
Serious end-organ complications of preeclampsia	
Eclampsia	Occurrence of generalised convulsions during pregnancy, labour or within 42 days of delivery in the absence of epilepsy or another condition predisposing to convulsions
Stroke	Hemiparesis and/or blindness developed during pregnancy or in the 42 days postpartum lasting greater than 48 hours
Coma	Prolonged unconsciousness ≥12 hours
Antepartum hemorrhage	Vaginal bleeding ≥ 15 mL with or without pain before the onset of labour
Disseminated intravascular coagulation	Abnormal bleeding from mucosa (mouth and/or ears)
Other major causes of maternal mortality/morbidity	
Obstetric sepsis	In the community, defined as fever and one of: abdominal/uterine tenderness, foul smelling vaginal discharge/lochia, productive cough and shortness of breath, dysuria or flank pain, headache and neck stiffness
Vesicovaginal or rectovaginal fistula	Continuous loss of urine and/or faeces after delivery
Life-saving interventions	
Cardiopulmonary resuscitation	A set of emergency procedures including chest compressions and lung ventilation applied in cardiac arrest victims
Mechanical ventilation	Intubation and ventilation not related to Cesarean section or anaesthesia
Blood transfusion	Of one or more units
Interventions for major postpartum hemorrhage	Brace sutures, external and internal uterine compression, anti-shock garment use, internal iliac artery ligation and/or hysterectomy with or without transfusion
Dialysis	Hemodialysis and/or peritoneal dialysis
**PERINATAL**	
**Perinatal and late neonatal death**	One or more of stillbirth, early neonatal mortality, or late neonatal mortality per 1,000 identified pregnancies
Stillbirth	Fetal death at ≥20+0 weeks' gestation and/or ≥500g birthweight

**CLIP primary outcome components**	**Definition**
Early neonatal mortality	Newborn death at 0-7 days of postnatal life
Late neonatal mortality	Newborn death at 8-28 days of postnatal life
**Neonatal morbidity**	Occurrence of a primary neonatal morbidity during d 0-28 of postnatal life per 1,000 identified pregnancies
Feeding difficulty	Including inability to suckle normally or latch on to the mother's breast to feed even if the mother's milk is not let down
Breathing difficulty	Including grunting and in-drawing of the abdomen under the ribs
Seizure	Occurrence of any seizure event (fits)
Lethargy	Baby not appearing normally wakeful after activities such as feeding or sleeping
Coma	Not medically induced period of unconsciousness of any length
Hypothermia	Cold to touch
Umbilical cord infection	Characterized by discharge from and redness around the umbilical stump
Skin infection	Any appearance of abnormally red, black, swollen and blistered skin with pus
Bleeding	From anywhere
Jaundice	Yellow skin and eyes
Central nervous system related morbidity	Abnormal amount of vomiting as defined by the parents or caregiver with bulging or sunken fontanelle

Table S3: Summary of pregnancies included in each analysis (median [interquartile range] and N (%), unless otherwise specified)

Characteristics of pregnancies in the analysis	All women		Hypertensive women	
	**Received antenatal POM visits and followed-up to primary outcome (N=20,819)**	**Eligible** (received ≥2 antenatal POM visits and followed-up to primary outcome) **(N=17,770)**	**Developed hypertension before birth (N=1236)**	**Eligible** (Developed hypertension before birth and had at least one subsequent antenatal POM visit) **(N = 751)**
**Characteristics at enrolment**				
Maternal age (yr)	25 [22, 30]	25 [22, 30]	25 [22, 30]	25 [22, 30]
GA enrolment(wk)	17.0 [11.1, 24.1]	16.4 [11.0, 22.8]	15 [9.86, 22.78]	15 [9.87, 22.8]
Nulliparous	5843 (28.1%)	5032 (28.3%)	410 (33.2%)	230 (30.6%)
Basic education	8375 (40.2%)	7231 (40.7%)	556 (45%)	337 (44.9%)
**Post-enrolment BP**				
BP level				
Mean sBP	106.0 [100.4, 112]	105.9 [100.5, 111.7]	117.7 [111.0,125.3]	118.5 [111.0, 127.3]
Mean dBP	66.6 [62.5, 71.0]	66.4 [62.5, 70.7]	76.9 [71.5,83.3]	78.0 [72.1, 85.3]
BP variability				
SD of sBP	6.27 [4.19, 8.77]	6.27 [4.19, 8.77]	11.68 [8.04, 16.09]	11.56 [7.39, 16.00]
ARV of sBP	6.67 [4.43, 9.83]	6.67 [4.43, 9.83]	10.00 [7.00, 14.33]	10.11 [7.00, 14.67]
SD of dBP	5.28 [3.54, 7.50]	5.28 [3.54, 7.50]	10.61 [8.08, 13.90]	10.56 [7.68, 13.99]
ARV of dBP	5.67 [3.75, 8.20]	5.67 [3.75, 8.20]	9.20 [6.40, 13.69]	9.30 [6.49, 14.00]
**Outcomes**				
Progression to hypertension	2084 (10%)	1893 (10.7%)	-	-
Primary outcome	4870 (23.4%)	4085 (23%)	378 (30.6%)	228 (30.4%)
Maternal composite	2052 (9.9%)	1696 (9.5%)	144 (11.7%)	90 (12%)
Perinatal composite	3484 (16.7%)	2929 (16.5%)	294 (23.8%)	177 (23.6%)

ARV (average real variability), BP (blood pressure), dBP (diastolic blood pressure), GA (gestational age), sBP (systolic blood pressure), SD (standard deviation)

Table S4: Relationship amongst ALL WOMEN, between mean systolic and diastolic blood pressure LEVEL and CLIP outcomes*

**Outcomes**	**Mean sBP**		**Mean dBP**	
	OR (95%CI)†	p-value	OR (95%CI)†	p-value
**Primary composite**	1.05 (1.03, 1.07)	<0.001	1.08 (1.06, 1.11)	<0.001
Maternal events				
Composite	1.04 (1.01, 1.07)	0.017	1.05 (1.01, 1.09	0.010
Mortality	1.15 (0.99, 1.35)	0.071	1.15 (0.93, 1.42)	0.198
Morbidity	1.03 (1.00, 1.06)	0.037	1.05 (1.01, 1.09)	0.012
Perinatal events				
Composite	1.06 (1.03, 1.08)	<0.001	1.10 (1.07, 1.14)	<0.001
Stillbirth	1.18 (1.14, 1.23)	<0.001	1.24 (1.18, 1.3)	<0.001
Early neonatal death	1.03 (0.98, 1.08)	0.254	1.09 (1.02, 1.15)	0.008
Late neonatal death	1.05 (0.96, 1.16)	0.272	1.17 (1.04, 1.31)	0.007
Neonatal morbidity	1.00 (0.98, 1.03)	0.753	1.02 (0.99, 1.06)	0.195

OR (odds ratio), CI (confidence interval), GA (gestational age), SD (standard deviation)

** OR and 95% CI that do not cross 1.00 are highlighted in yellow for clarity.*
† OR and 95% CI results are presented for a 5mm Hg increase in blood pressure and are estimated from mixed effects logistic regression adjusted for country and cluster (each as a random effect), GA at enrolment, maternal age, parity and maternal education.

Table S5: Relationship amongst ALL WOMEN, between systolic and diastolic blood pressure VARIABILITY and CLIP outcomes (represented graphically in Figure 3)*

**Outcomes**	**Systolic blood pressure**				**Diastolic blood pressure**			
	SD OR (95%CI)†	p-value	ARV OR (95%CI)†	p-value	SD OR (95%CI)†		ARV OR (95%CI)†	p-value
**Hypertension**	1.78 [1.70, 1.88]	< 0.001	1.40 (1.34, 1.47)	< 0.001	2.15 (2.05, 2.27)	< 0.001	1.65 (1.57, 1.73)	< 0.001
**Primary composite**	1.10 (1.06, 1.14)	< 0.001	1.06 (1.02, 1.10)	0.003	1.07 (1.03, 1.11)	< 0.001	1.06 (1.02, 1.09)	0.003
Maternal events								
Composite	1.08 (1.03, 1.14)	0.003	1.04 (0.99, 1.10)	0.127	1.08 (1.02, 1.13)	0.004	1.05 (1.00, 1.11)	0.037
Mortality	1.23 (0.96, 1.59)	0.097	1.19 (0.94, 1.50)	0.127	1.39 (1.14, 1.70)	0.001	1.35 (1.14, 1.60)	0.001
Morbidity	1.08 (1.02, 1.13)	0.007	1.04 (0.99, 1.09)	0.156	1.08 (1.02, 1.13)	0.005	1.05 (1.00, 1.10)	0.050
Perinatal events								
Composite	1.08 (1.04, 1.13)	< 0.001	1.06 (1.02, 1.11)	0.003	1.05 (1.01, 1.09)	0.026	1.05 (1.01, 1.10)	0.011
Stillbirth	1.12 (1.04, 1.20)	0.002	1.12 (1.05, 1.20)	0.001	1.10 (1.03, 1.18)	0.006	1.12 (1.05, 1.20)	0.001
Early NND	0.98 (0.90, 1.07);	0.686	1.00 (0.92, 1.09)	0.950	0.98 (0.90, 1.07)	0.644	1.00 (0.92, 1.09)	0.920
Late NND	1.11 (0.95, 1.30)	0.179	1.07 (0.91, 1.25)	0.414	1.03 (0.88, 1.20)	0.707	1.03 (0.89, 1.20)	0.686
Neonatal morbidity	1.09 (1.04, 1.15)	< 0.001	1.05 (1.00, 1.10)	0.069	1.05 (1.00, 1.10)	0.074	1.02 (0.97, 1.08)	0.078

ARV (average real variability), CI (confidence interval), NND (neonatal death), OR (odds ratio), SD (standard deviation)

** OR and 95% CI that do not cross 1.00 are highlighted in yellow for clarity.*
† OR and 95% CI results are presented for a SD increase in corresponding measure of BP variability amongst women included in analysis, and are estimated from mixed effects logistic regression adjusted for country and cluster (each as a random effect), BP level, GA at enrolment, maternal age, parity and maternal education. The cells highlighted in yellow represent significant effects at the p<0.05 level.

Table S6: Sensitivity analyses - relationship amongst ALL WOMEN, between systolic and diastolic blood pressure VARIABILITY and CLIP outcomes, excluding blood pressure measurements taken 7, 14, 21 and 28 days prior to delivery*

	**Blood pressure**									
**Outcomes**	**All measurements**		**Excluding 7 days before**		**Excluding 14 days before**		**Excluding 21 days before**		**Excluding 28 days before**	
	SD OR (95%CI)†; p-value	ARV OR (95%CI)†; p-value	SD OR (95%CI)†; p-value	ARV OR (95%CI)†; p-value	SD OR (95%CI)†; p-value	ARV OR (95%CI)†; p-value	SD OR (95%CI)†; p-value	ARV OR (95%CI)†; p-value	SD OR (95%CI)†; p-value	ARV OR (95%CI)†; p-value
**Systolic blood pressure**										
Primary composite	1.10 (1.06, 1.14) < 0.001	1.06 (1.02,1.10); 0.003	1.04 (1.01,1.09); 0.025	1.04 (1.00,1.08); 0.033	1.03 (0.99,1.07); 0.189	1.03 (0.99,1.07); 0.135	1.02 (0.98,1.06); 0.254	1.02 (0.98,1.07); 0.247	1.02 (0.98,1.07); 0.253	1.02 (0.98,1.06); 0.427
**Maternal events**										
Composite	1.08 (1.03,1.14); 0.003	1.04 (0.99,1.10); 0.127	1.02 (0.97, 1.08); 0.401	1.02 (0.96,1.07); 0.527	1.01 (0.96,1.07); 0.718	1.02 (0.96,1.08); 0.51	1.01 (0.95,1.07); 0.781	1.01 (0.95,1.07); 0.816	0.99 (0.94,1.05); 0.792	0.99 (0.93,1.05); 0.662
Mortality	1.23 (0.96,1.59); 0.104	1.19 (0.94,1.50); 0.152	1.27 (0.94,1.71); 0.118	1.25 (0.96,1.62); 0.103	1.29 (0.96,1.73); 0.089	1.26 (0.97,1.63); 0.090	1.28 (0.96,1.71); 0.092	1.22 (0.93,1.59); 0.155	1.25 (0.87,1.79); 0.22	1.14 (0.79,1.66); 0.48
Morbidity	1.08 (1.02,1.13); 0.005	1.04 (0.99,1.09); 0.156	1.02 (0.97, 1.08); 0.472	1.01 (0.96,1.07); 0.612	1.01 (0.95,1.06); 0.823	1.01 (0.96,1.07); 0.602	1.00 (0.95,1.06); 0.896	1.00 (0.95,1.06); 0.923	0.99 (0.93,1.05); 0.757	0.99 (0.93,1.05); 0.657
**Perinatal events**										
Composite	1.08 (1.04,1.13); < 0.001	1.06 (1.02,1.11); 0.003	1.04 (1.00,1.09); 0.056	1.05 (1.01,1.1); 0.023	1.03 (0.99,1.08); 0.183	1.04 (0.99,1.08); 0.095	1.03 (0.98,1.07); 0.24	1.03 (0.99,1.08); 0.137	1.03 (0.99,1.08); 0.146	1.03 (0.99,1.08); 0.156
Stillbirth	1.12 (1.04,1.20); 0.002	1.12 (1.05,1.20); 0.001	1.07 (1.00,1.16); 0.064	1.1 (1.02,1.18); 0.011	1.06 (0.98,1.14); 0.151	1.08 (1.01,1.17); 0.031	1.07 (0.99,1.15); 0.097	1.09 (1.02,1.18); 0.018	1.03 (0.95,1.12); 0.454	1.07 (0.99,1.15); 0.100
Early neonatal death	0.98 (0.90,1.07); 0.686	1.00 (0.92,1.09); 0.950	0.99 (0.90,1.09); 0.841	1.01 (0.92,1.10); 0.886	0.98 (0.90,1.08); 0.749	0.99 (0.90,1.09); 0.846	0.97 (0.88,1.06); 0.483	0.97 (0.88,1.07); 0.584	1.00 (0.91,1.10); 0.987	0.99 (0.90,1.09); 0.841

	Blood pressure									
**Outcomes**	**All measurements**		**Excluding 7 days before**		**Excluding 14 days before**		**Excluding 21 days before**		**Excluding 28 days before**	
	SD OR (95%CI)†; p-value	ARV OR (95%CI)†; p-value	SD OR (95%CI)†; p-value	ARV OR (95%CI)†; p-value	SD OR (95%CI)†; p-value	ARV OR (95%CI)†; p-value	SD OR (95%CI)†; p-value	ARV OR (95%CI)†; p-value	SD OR (95%CI)†; p-value	ARV OR (95%CI)†; p-value
Late neonatal death	1.11 (0.95, 1.3); 0.179	1.07 (0.91,1.25); 0.414	1.02 (0.86,1.21); 0.821	0.99 (0.83,1.18); 0.885	0.97 (0.81,1.17); 0.777	0.94 (0.78,1.14); 0.541	0.96 (0.79,1.15); 0.633	0.93 (0.77,1.13); 0.459	0.94 (0.78,1.15); 0.555	0.92 (0.75,1.12); 0.413
Morbidity	1.09 (1.04,1.15); < 0.001	1.05 (1.00,1.10); 0.069	1.04 (0.98,1.09); 0.187	1.03 (0.98,1.09); 0.237	1.03 (0.97,1.08); 0.331	1.02 (0.97,1.08); 0.393	1.02 (0.96,1.07); 0.561	1.01 (0.96,1.07); 0.644	1.04 (0.98,1.10); 0.164	1.03 (0.97,1.09); 0.395
**Diastolic blood pressure**										
**Primary composite**	1.07 (1.03,1.11); < 0.001	1.06 (1.02,1.09); 0.003	1.04 (1.00,1.08); 0.05	1.04 (1.01,1.08); 0.022	1.04 (1.00,1.08); 0.062	1.04 (1.00,1.08); 0.043	1.03 (0.99,1.07); 0.095	1.04 (1.00,1.08); 0.066	1.03 (0.99,1.07); 0.152	1.03 (0.99,1.07); 0.219
**Maternal events**										
Composite	1.08 (1.02,1.13); 0.004	1.05 (1.00,1.11); 0.037	1.04 (0.99,1.10); 0.120	1.04 (0.99,1.10); 0.14	1.04 (0.99,1.10); 0.106	1.04 (0.99,1.10); 0.117	1.05 (0.99,1.11); 0.077	1.04 (0.98,1.10); 0.170	1.03 (0.97,1.09); 0.32	1.02 (0.96,1.08); 0.492
Mortality	1.39 (1.14,1.70); 0.001	1.35 (1.14,1.60); 0.001	1.40 (1.12,1.77); 0.004	1.35 (1.10,1.65); 0.004	1.41 (1.13,1.77); 0.003	1.36 (1.11,1.66); 0.003	1.38 (1.08,1.76); 0.009	1.32 (1.08,1.63); 0.008	1.42 (1.09,1.86); 0.010	1.36 (1.05,1.75); 0.018
Morbidity	1.08 (1.02,1.13); 0.005	1.05 (1.00,1.10); 0.050	1.04 (0.99,1.10); 0.15	1.04 (0.98,1.09); 0.171	1.04 (0.99,1.10); 0.136	1.04 (0.99,1.10); 0.15	1.05 (0.99,1.11); 0.101	1.04 (0.98,1.09); 0.21	1.03 (0.97,1.09); 0.357	1.02 (0.96,1.08); 0.517
**Perinatal events**										
Composite	1.05 (1.01,1.09); 0.026	1.05 (1.01,1.10); 0.011	1.04 (0.99,1.08); 0.103	1.05 (1.01,1.10); 0.015	1.03 (0.99,1.08); 0.166	1.04 (1.00,1.09); 0.044	1.03 (0.98,1.07); 0.208	1.05 (1.00,1.09); 0.045	1.02 (0.98,1.07); 0.339	1.03 (0.99,1.08); 0.186
Stillbirth	1.10 (1.03,1.18); 0.006	1.12 (1.05,1.20); 0.001	1.11 (1.03,1.19); 0.005	1.13 (1.05,1.21); 0.001	1.11 (1.03,1.19); 0.007	1.12 (1.05,1.21); 0.001	1.10 (1.02,1.19); 0.01	1.12 (1.05,1.21); 0.002	1.08 (1.00,1.16); 0.06	1.10 (1.02,1.19); 0.01

	Blood pressure									
**Outcomes**	**All measurements**		**Excluding 7 days before**		**Excluding 14 days before**		**Excluding 21 days before**		**Excluding 28 days before**	
	SD OR (95%CI)†; p-value	ARV OR (95%CI)†; p-value	SD OR (95%CI)†; p-value	ARV OR (95%CI)†; p-value	SD OR (95%CI)†; p-value	ARV OR (95%CI)†; p-value	SD OR (95%CI)†; p-value	ARV OR (95%CI)†; p-value	SD OR (95%CI)†; p-value	ARV OR (95%CI)†; p-value
Early neonatal death	0.98 (0.90,1.07); 0.644	1.00 (0.92,1.09); 0.92	0.99 (0.90,1.08); 0.794	1.01 (0.92,1.10); 0.871	1.00 (0.91,1.09); 0.998	1.00 (0.92,1.1); 0.92	1 (0.92,1.10); 0.959	1.02 (0.93,1.11); 0.679	1.00 (0.91,1.1); 0.974	1.01 (0.92,1.11); 0.808
Late neonatal death	1.03 (0.88,1.20); 0.707	1.03 (0.89,1.20); 0.686	0.95 (0.79,1.13); 0.55	0.97 (0.81,1.15); 0.696	0.94 (0.78,1.12); 0.49	0.96 (0.80,1.14); 0.622	0.91 (0.76,1.10); 0.345	0.94 (0.78,1.13); 0.501	0.83 (0.67,1.02); 0.082	0.86 (0.70,1.06); 0.156
Morbidity	1.05 (1.00,1.10); 0.074	1.02 (0.97,1.08); 0.357	1.01 (0.96,1.06); 0.771	1.02 (0.96,1.07); 0.553	1.00 (0.95,1.05); 0.942	1.01 (0.95,1.06); 0.765	0.99 (0.94,1.05); 0.744	1.00 (0.94,1.06); 0.955	0.99 (0.94,1.05); 0.741	0.99 (0.93,1.05); 0.711

ARV (average real variability), CI (confidence interval), OR (odds ratio), SD (standard deviation)

** OR and 95% CI that do not cross 1.00 are highlighted in yellow for clarity.*
† OR and 95% CI results are presented for a SD increase in blood pressure and are estimated from mixed effects logistic regression adjusted for country and cluster (each as a random effect), BP level, GA at enrolment, maternal age, parity and maternal education. The cells highlighted in yellow represent significant effects at the p<0.05 level.

Table S7: Sensitivity analyses - relationship amongst ALL WOMEN, between systolic and diastolic blood pressure VARIABILITY and CLIP outcomes, adjusting for the last BP value before birth*

**Outcomes**	**Systolic blood pressure**				**Diastolic blood pressure**			
	SD OR (95%CI)†	p-value	ARV OR (95%CI)†	p-value	SD OR (95%CI)†	p-value	ARV OR (95%CI)†	p-value
**Primary composite**	1.08 (1.04, 1.12)	< 0.001	1.05 (1.01, 1.09)	0.013	1.06 (1.02, 1.10)	0.002	1.05 (1.01, 1.09)	0.009
Maternal events								
Composite	1.04 (0.98, 1.10)	0.184	1.02 (0.97, 1.08)	0.426	1.05 (1.00, 1.11)	0.072	1.04 (0.99, 1.09)	0.141
Mortality	1.23 (0.96, 1.59)	0.102	1.12 (0.89, 1.42)	0.321	1.47 (1.20, 1.80)	< 0.001	1.39 (1.17, 1.65)	<0.001
Morbidity	1.03 (0.97, 1.09)	0.304	1.02 (0.96, 1.07)	0.536	1.05 (0.99, 1.10)	0.105	1.03 (0.98, 1.09)	0.186
Perinatal events								
Composite	1.08 (1.03, 1.13)	0.001	1.06 (1.02, 1.11)	0.006	1.05 (1.01, 1.10)	0.017	1.05 (1.01, 1.10)	0.010
Stillbirth	1.14 (1.06, 1.23)	< 0.001	1.12 (1.05, 1.20)	0.001	1.12 (1.04, 1.21)	0.002	1.13 (1.05, 1.21)	<0.001
Early neonatal death	0.98 (0.89, 1.08)	0.656	1.00 (0.92, 1.09)	0.978	1 (0.92, 1.09);	0.958	1.01 (0.93, 1.10)	0.920
Late neonatal death	1.11 (0.94, 1.32)	0.205	1.07 (0.91, 1.25)	0.416	1.01 (0.86, 1.20)	0.869	1.03 (0.88, 1.19)	0.686
Neonatal morbidity	1.07 (1.01, 1.13)	0.018	1.04 (0.98, 1.09)	0.168	1.04 (0.98, 1.09)	0.208	1.02 (0.97, 1.07)	0.489

ARV (average real variability), CI (confidence interval), OR (odds ratio), SD (standard deviation)

** OR and 95% CI that do not cross 1.00 are highlighted in yellow for clarity.*
†OR and 95% CI results are presented for a SD increase in corresponding measure of BP variability, and are estimated from mixed effects logistic regression adjusted for country and cluster (each as a random effect), BP level, last BP measurement prior to delivery, GA at enrolment, maternal age, parity and maternal education.

Table S8: Sensitivity analyses - Relationship amongst ALL WOMEN, between systolic and diastolic blood pressure VARIABILITY and CLIP outcomes, excluding the N=926 repeat pregnancies*

Outcomes	Systolic blood pressure				Diastolic blood pressure			
	SD odds ratio (95%CI)ł	p-value	ARV odds ratio (95%CI)ł	p-value	SD odds ratio (95%CI)ł		ARV odds ratio (95%CI)ł	p-value
**Hypertension**	2.12 (2.00,2.24)	< 0.001	1.54 (1.46, 1.62)	< 0.001	2.74 (2.54, 2.91)	< 0.001	1.87 (1.77, 1.98)	< 0.001
**Primary composite**	1.10 (1.06, 1.14)	< 0.001	1.06 (1.02, 1.10)	0.002	1.07 (1.03, 1.11)	< 0.001	1.05 (1.02, 1.09)	0.006
Maternal events								
Composite	1.08 (1.03, 1.14)	0.003	1.04 (0.99, 1.10)	0.127	1.07 (1.02, 1.13)	0.006	1.05 (1.00, 1.10)	0.058
Mortality	1.24 (0.96,1.60)	0.101	1.19 (0.94,1.50)	0.150	1.40 (1.15, 1.71)	0.001	1.35 (1.14, 1.60)	0.001
Morbidity	1.08 (1.02,1.14)	0.007	1.04 (0.99,1.10)	0.142	1.07 (1.02, 1.13)	0.009	1.05 (1.00, 1.10)	0.076
Perinatal events								
Composite	1.01 (1.04, 1.14)	< 0.001	1.07 (1.03, 1.12)	0.002	1.05 (1.01, 1.10)	0.025	1.05 (1.01, 1.10)	0.012
Stillbirth	1.13 (1.05, 1.22)	0.001	1.13 (1.05, 1.21)	0.001	1.10 (1.03, 1.19)	0.008	1.12 (1.05, 1.20)	0.001
Early neonatal death	1.00 (0.91, 1.09)	0.959	1.02 (0.93, 1.11)	0.661	0.97 (0.88, 1.06)	0.47	0.99 (0.90, 1.08)	0. 794
Late neonatal death	1.12 (0.94, 1.32)	0.197	1.08 (0.92, 1.27)	0.332	1.03 (0.87, 1.22)	0.73	1.03 (0.88, 1.21)	0.683
Neonatal morbidity	1.10 (1.04, 1.15)	< 0.001	1.05 (1.00, 1.11)	0.071	1.05 (1.00, 1.11)	0.056	1.03 (0.97, 1.08)	0.348

OR (odds ratio), SD (standard deviation)

** OR and 95% CI that do not cross 1.00 are highlighted in yellow for clarity.*

ł OR and 95% CI results are presented for a SD increase in corresponding measure of BP variability, and are estimated from mixed effects logistic regression adjusted for country and cluster (each as a random effect), BP level, GA at enrolment, maternal age, parity and maternal education. The cells highlighted in yellow represent significant effects at the p<0.05 level.

Table S9: Sensitivity analyses - relationship amongst ALL WOMEN, between systolic and diastolic blood pressure VARIABILITY and progression to hypertension, incorporating the diagnosis from trial surveillance*

Outcomes	Systolic blood pressure				Diastolic blood pressure			
	SD OR (95%CI)†	p-value	ARV OR (95%CI)†	p-value	SD OR (95%CI)†		ARV OR (95%CI)†	p-value
**Hypertension**	1.78 (1.70, 1.88)	<0.001	1.40 (1.34, 1.47)	< 0.001	2.15 (2.05, 2.27)	< 0.001	1.65 (1.57, 1.73)	< 0.001

ARV (average real variability), CI (confidence interval), OR (odds ratio), SD (standard deviation)

** OR and 95% CI that do not cross 1.00 are highlighted in yellow for clarity.*
†OR and 95% CI results are presented for a SD increase in corresponding measure of BP variability, and are estimated from mixed effects logistic regression adjusted for country and cluster (each as a random effect), BP level, GA at enrolment, maternal age, parity and maternal education.

Table S10: Relationship amongst ONLY HYPERTENSIVE WOMEN between blood pressure VARIABILITY and maternal and perinatal outcomes*

Outcomes	**Systolic blood pressure**				**Diastolic blood pressure**			
	SD OR (95%CI)†	p-value	ARV OR (95%CI)†	p-value	SD OR (95%CI)†		ARV OR (95%CI)†	p-value
**Primary composite**	1.32 (1.12, 1.56)	0.001	1.14 (0.96, 1.35)	0.13	1.21 (1.03, 1.43)	0.023	1.09 (0.92, 1.29)	0.294
Maternal events								
Composite	1.26 (1.02, 1.56)	0.035	1.02 (0.82, 1.28)	0.858	1.28 (0.98, 1.67)	0.067	1.20 (0.97, 1.48)	0.095
Mortality	2.07 (1.14, 3.76)	0.017	1.69 (1.05, 2.71)	0.029	1.99 (0.74, 5.37)	0.174	1.97 (0.94, 4.12)	0.073
Morbidity	1.23 (0.98, 1.53)	0.068	0.98 (0.77, 1.25)	0.874	1.40 (1.12, 1.76)	0.003	1.21 (0.97, 1.50)	0.089
Perinatal events								
Composite	1.19 (0.93, 1.52)	0.164	1.13 (0.95, 1.36)	0.168	1.09 (0.91, 1.31)	0.355	1.0 (0.82, 1.21)	0.992
Stillbirth	1.08 (0.76, 1.52)	0.668	1.05 (0.83, 1.35)	0.67	1.05 (0.80, 1.36)	0.735	0.95 (0.71, 1.26)	0.717
Early NND	1.32 (0.71, 2.42)	0.379	1.03 (0.74, 1.44)	0.855	1.13 (0.80, 1.61)	0.485	1.03 (0.71, 1.50)	0.884
Late NND	1.24 (0.98, 1.56)	0.071	1.39 (0.85, 2.25)	0.186	0.8 (0.40, 1.62)	0.538	0.99 (0.49, 1.97)	0.966
Morbidity	1.21 (0.89, 1.66)	0.230	1.29 (1.02, 1.63)	0.036	1.14 (0.89, 1.45)	0.302	1.13 (0.89, 1.45)	0.319

ARV (average real variability), CI (confidence interval), GA (gestational age), NND (neonatal death), OR (odds ratio), SD (standard deviation)
** OR and 95% CI that do not cross 1.00 are highlighted in yellow for clarity.*
† OR and 95% CI results are presented for a SD increase in corresponding measure of BP variability amongst women included in analysis, and are estimated from mixed effects logistic regression adjusted for country and cluster (each as a random effect), BP level, GA at enrolment, maternal age, parity and maternal education.
